# Downregulated Dopamine Receptor 2 and Upregulated Corticotrophin Releasing Hormone in the Paraventricular Nucleus Are Correlated With Decreased Glucose Tolerance in Rats With Bilateral Substantia Nigra Lesions

**DOI:** 10.3389/fnins.2019.00751

**Published:** 2019-07-23

**Authors:** Li Zhou, Xue-Rui Ran, Feng Hong, Guang-Wen Li, Jin-Xia Zhu

**Affiliations:** ^1^Department of Physiology and Pathophysiology, School of Basic Medical Sciences, Capital Medical University, Beijing, China; ^2^Xinxiang Key Laboratory of Molecular Neurology, Department of Human Anatomy, Xinxiang Medical University, Xinxiang, China

**Keywords:** glucose metabolism, Parkinson’s disease, dopamine receptor, corticotrophin releasing hormone, hypothalamic-pituitary-adrenal axis

## Abstract

Patients with Parkinson’s disease (PD) have a high prevalence of glucose metabolism abnormalities. However, the mechanism underlying these symptoms remains unclear. The hypothalamic-pituitary-adrenal (HPA) axis is the major neuroendocrine axis that regulates homeostasis in mammals, including glucose metabolism. Corticotrophin releasing hormone (CRH), which is synthesized in the paraventricular nucleus (PVN) of the hypothalamus, plays an important role in the regulation of blood glucose levels via the HPA axis. Our previous studies have reported that PVN neurons express numerous dopamine receptors (DRs) and accept direct projections from the substantia nigra (SN). We hypothesize that damage to dopaminergic neurons in the SN might influence the blood glucose level through the HPA system. Rats with bilateral SN lesions induced by 6-hydroxydopamine (6-OHDA) (referred to as 6-OHDA rats) were used to investigate alterations in the levels of blood glucose, CRH, and factors related to the HPA axis and to explore possible mechanisms. Blood glucose levels were detected at different time points after the glucose solution was intraperitoneally administered. CRH and DRs in the PVN were evaluated by immunofluorescence and western blot analysis. Adrenocorticotropic hormone (ACTH) in the pituitary and plasma corticosterone (CORT) was evaluated by radioimmunoassay (RIA). The results showed that 6-OHDA rats exhibited significantly decreased tyrosine hydroxylase (TH) in the SN and decreased glucose tolerance at 6 weeks, but not at 4 weeks. In the PVN, dopamine receptor 2 (D2) was expressed on CRH-positive neurons, and D2-positive neurons were surrounded by TH-positive fibers. Additionally, the expression of CRH was upregulated, whereas the expression of D2 and TH were downregulated in 6-OHDA rats compared with control rats. In D2 knock-out mice, the significantly enhanced expression of CRH and reduced expression of D2 were detected in the PVN. Furthermore, RIA revealed increased ACTH in the pituitary and elevated CORT in the blood. In summary, the present study suggests that the dopaminergic neurons in the SN are involved in the regulation of body glucose metabolism through CRH neurons that express D2 in the hypothalamic PVN. SN lesions decrease glucose tolerance mainly by downregulating D2 and upregulating CRH in the PVN through the HPA neuroendocrine system.

## Introduction

Parkinson’s disease (PD) is a common neurodegenerative disorder characterized by the progressive loss of dopaminergic neurons in the substantia nigra (SN), which manifests both motor and non-motor symptoms (NMS) (Stern et al., 2012). While the classic motor features are due to the loss of nigrostriatal dopaminergic cells, the spectrum of NMS reflects a more complex etiology, including neuroendocrine and metabolic abnormalities (De Pablo-Fernandez et al., 2017). People realize that NMS play a tremendously important role in the management and even the diagnosis of the disorder (Pfeiffer, 2016). Here, we focused on the high prevalence of glucose metabolism abnormalities observed in patients with PD, which have been extensively studied (Lipman et al., 1974; Dunn et al., 2014; De Pablo-Fernandez et al., 2017; Biosa et al., 2018). It is important to elucidate the mechanism of impaired glucose metabolism in patients with PD for controlling and delaying the onset and progression of disease-related complications in the early stage of the disease. However, the underlying pathogenesis remains unclear.

The hypothalamic-pituitary-adrenal (HPA) axis is the major neuroendocrine axis that regulates homeostasis in mammals, including glucose metabolism (Si et al., 2015). Corticotrophin releasing hormone (CRH), which is synthesized in the paraventricular nucleus (PVN) of the hypothalamus, plays an important role in regulating blood glucose via the HPA axis (Lu et al., 2018). CRH is released into the hypophyseal portal capillaries in the median eminence and stimulates the secretion of adrenocorticotropic hormone (ACTH) in the pituitary gland. Then, ACTH arrives at the adrenal cortex through the systemic blood circulation and promotes the synthesis and secretion of glucocorticoid hormones, cortisol or corticosterone (CORT), in the adrenal cortex, participating in the regulation of blood sugar (Spencer and Deak, 2017).

Reduced PVN function has been reported in patients with PD (Mann and Yates, 1983; Jellinger, 1991). Our previous studies have shown that the PVN receives a direct projection from the SN by neural tracing technology (Wang et al., 2014), and a large number of dopamine receptors 1 and 2 (D1 and D2) are distributed throughout the PVN (Ran et al., 2019). We hypothesized that the CRH-positive neurons in the PVN express dopamine receptors (DRs) and that dopamine from the SN could influence the synthesis and secretion of CRH via DRs in the PVN, which in turn regulate the blood glucose level via the HPA system.

The present study aimed to explore the relationship between PD and glucose metabolism and its underlying mechanism by using a classic animal model established with the neurotoxin 6-hydroxydopamine (6-OHDA), which cannot cross the blood-brain barrier and therefore requires direct administration on the SN (Jackson-Lewis et al., 2012). Our findings provide direct evidence that the loss of dopamine in the SN leads to decreased glucose tolerance, which is correlated with the altered HPA neuroendocrine system.

## Materials and Methods

### Animals

Male Sprague–Dawley rats (Laboratory Animal Services Center of Capital Medical University, Beijing, China) that ranged in weight from 210 to 230 g (7∼8 weeks) were used. All animals were housed in animal care facilities at 22 ± 1°C on a 12:12 h light–dark cycle. Food and water were provided *ad libitum*.

Global D2 knock-out mice were generated and purchased from the Institute of Laboratory Animals Science, the Chinese Academy of Medical Sciences & Peking Union Medical College. Sex- and age-matched wild-type littermates were used as controls. All male mice used had a C57BL/6 background, were 8 weeks of age and were housed under the same environmental conditions as the rats.

Every procedure was approved by the Animal Care and Use Committee of Capital Medical University and was conducted according to the established guidelines of the National Institutes of Health (NIH, United States).

### 6-OHDA Animal Models

The animal model of PD was made by bilaterally microinjecting the SN with 6-OHDA (Sigma, United States) (referred to as 6-OHDA rats) as previously reported (Zheng et al., 2011). Briefly, all animals were anesthetized with an intraperitoneal (i.p.) injection of chloral hydrate (0.4 g/kg) and placed on a stereotaxic instrument. Two holes were drilled into the skull (coordinates: anterior-posterior (AP), −5.6 mm; medial-lateral (ML), ± 2.0 mm; dorsal-ventral (DV), −7.5 mm), and 6-OHDA (4 μg in μl of 0.9% saline containing 0.05% ascorbic acid) was bilaterally injected with a 10-μl Hamilton syringe. Control groups were injected with saline containing 0.2% ascorbic acid. Subsequent experiments were performed 4 and 6 weeks after 6-OHDA administration.

### ipGTT

At 4 and 6 weeks, the intraperitoneal glucose tolerance test (ipGTT) was performed in fasted (8 h) rats at approximately 16:00. The tips of the tails of the rats were cut (less than 1 mm) for blood collection. The first drop was discarded, and the second drop was used for the determination of blood glucose (time 0) using a glucometer (Accu-Chek Performa; Roche Diagnostics, GmbH, Mannheim, Germany). Immediately afterward, a 20% glucose solution prewarmed at 37°C (2 g/kg) was intraperitoneally injected, and blood samples were collected at 15, 30, 60, and 120 min for blood glucose measurements as previously described (Oh et al., 2013).

### Tissue Preparation

For brain sections, the animals were perfused through the left ventricle according to the previous method (Zhou et al., 2014). The brains were then quickly removed and kept in 4% paraformaldehyde for 24 h after fixation. After dehydration with 15 and 30% sucrose in 0.01 M PBS (pH 7.4), coronal frozen sections including the SN and PVN were cut to a thickness of 20 μm with a cryostat (Leica CM1950, Switzerland). The brain sections were air-dried overnight at room temperature and stored at −80°C.

For western blot analysis and radioimmunoassay, samples of the SN, hypothalamus, pituitary gland and adrenal gland were collected on ice and immediately frozen in liquid nitrogen. The tissues were stored at −80°C until further testing.

### IF and IHC Staining

For immunofluorescence (IF), as described in our previous report (Zhou et al., 2014). Briefly, after retrieving antigens by heating to 95°C–100°C in a beaker containing citrate buffer (0.01 M, pH 6.0) for 15 min, the sections were incubated with 10% normal goat serum for 1 h at room temperature. Then, the sections were incubated with primary antibodies (Table 1) at 4°C overnight, and subsequently incubated with secondary antibodies (Table 2) for 1 h at room temperature. Photomicrographs were obtained using a confocal microscope (Olympus, FV1000).

**TABLE 1:** First antibodies used in this study.

**Antigen**	**Antibody**	**Dilution**		**Source/catalog no.**
			
		**IHC/IF**	**Western blot**	
TH	Mouse monoclonal	1:5000	1:10000	Sigma/T1299
D1	Rabbit polyclonal	1:100	1:500	Alomone/ADR-001
D2	Rabbit polyclonal	1:100	1:500	Alomone/ADR-002
D2	Mouse monoclonal	1:100	1:500	Santa Cruz/sc-5303
CRH	Rabbit polyclonal	1:100	1:500	Cloud-clone corp/PAA853Hu01
GAPDH	Rabbit polyclonal	N/A	1:5000	Sigma/G9545

*TH, tyrosine hydroxylase; D1, dopamine 1 receptor; D2, dopamine 2 receptor; CRH, corticotrophin releasing hormone.*

**TABLE 2:** Secondary antibodies used in this study.

**Antigen**	**Conjugation**	**Dilution**	**Source/Catalog no.**
anti-mouse IgG (IHC)	HRP	HRP	ZSGB-Bio/PV-9002
Goat anti-mouse IgG (IF)	Alexa Fluor 488	1:1000	Invitrogen/A11017
Goat anti-mouse IgG (IF)	Alexa Fluor 594	1:1000	Invitrogen/A11020
Donkey anti-rabbit IgG (IF)	Alexa Fluor 488	1:1000	Invitrogen/A21206
Donkey anti-rabbit IgG (IF)	Alexa Fluor 594	1:1000	Invitrogen/A21207
Goat anti-rabbit IgG (WB)	IRDye800	1:10000	Rockland/611-132-122
Sheep anti-mouse IgG (WB)	IRDye800	1:10000	Rocklane/610-632-002

For immunohistochemistry (IHC), in contrast with IF, the slices were quenched in endogenous peroxidase using 0.3% H_2_O_2_ before antigen retrieval. After incubation with primary antibody (Table 1) at 4°C overnight, immunostaining was performed using a PV-9002 Polymer Detection System (ZSGB-Bio, China) with diaminobenzidine according to the manufacturer’s protocol. The sections were photographed with a light microscope (Nikon E80i, Japan).

### Western Blot Analysis

The frozen brain tissues were homogenized in 300 μl of cold lysis buffer supplemented with protease inhibitors for protein extraction. Proteins (100 μg) were separated by 10% SDS-PAGE and transferred to a nitrocellulose membrane. After blocking with 5% skim milk in PBS for 1 h, the membranes were incubated with primary antibodies (Table 1) overnight at 4°C. Then, the membranes were incubated with the appropriate secondary antibodies (Table 2) for 1 h at room temperature. After the final wash, the membrane was scanned and quantified with an Odyssey Infrared Image system (LI-COR, United States). The integrated intensity of the bands was analyzed by Odyssey software (version 3.0).

### Radioimmunoassay

Under anesthesia, blood samples were collected into cold EDTA tubes from the heart and centrifuged at 3000 rpm (4°C) for 10 min to isolate the plasma. The supernatant was transferred to new Eppendorf tubes and stored at −80°C. Then, homogenates of hypothalamus, pituitary gland and adrenal gland samples were also centrifuged to obtain the supernatant. ACTH and CORT levels in the blood samples and brain tissue homogenates were detected by Beijing Furunruize Biotechnology Co., Ltd.

### Statistical Analysis

The results are presented as the mean ± SEM from at least three experiments. Statistical analyses were performed using the unpaired Student’s *t* test (GraphPad Software 5.0, La Jolla, CA, United States). Differences were considered significant at *p* < 0.05.

## Results

### SN Lesions and Attenuated Glucose Tolerance in 6-OHDA-Induced Rats

**FIGURE 1:**
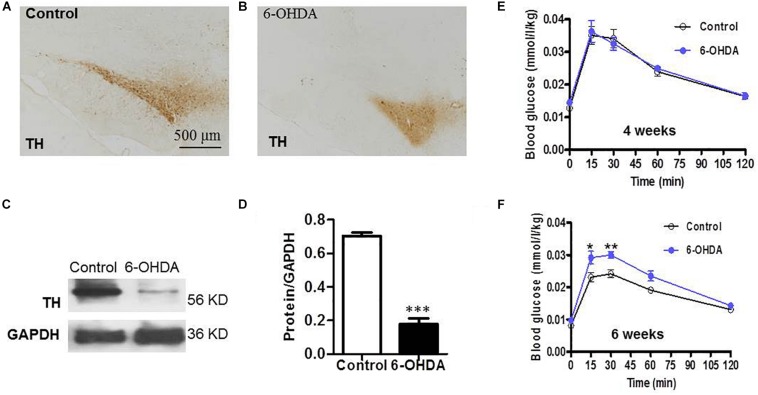
SN lesions and attenuated glucose tolerance in 6-OHDA-induced rats. **(A)** (Control group) and **(B)** (6-OHDA group): Representative images of TH-IR neurons in the SN, Scale bar, 500 μm; **(C)** Representative western blot of TH in the SN of control and 6-OHDA rats. **(D)** Summary histogram of C, ^*^p < 0.05, ^∗∗^*p* < 0.01, and ^∗∗∗^*p* < 0.001 (*n* = 6). **(E,F)** Blood glucose values during the ipGTT on the 4th and 6th weeks, respectively, (*n* = 10).

As a rate-limiting enzyme in catecholamine synthesis, tyrosine hydroxylase (TH) is normally used as a marker for intrinsic catecholaminergic neurons (Zheng and Travagli, 2007). The majority of TH-immunoreactive (TH-IR) neurons in the SN are dopaminergic; thus, TH-IR neurons were observed to indirectly detect the dopaminergic neurons in the SN. Six weeks after injecting 6-OHDA into the SN, IHC analysis showed that the field number of TH-IR neurons in the SN was significantly reduced in 6-OHDA rats (Figure 1B) compared with control rats (Figure 1A). Western blot analysis showed that the protein level of TH in the SN was markedly decreased in 6-OHDA rats compared with control rats, from 0.70 ± 0.02 to 0.18 ± 0.04 (*n* = 6, *p* < 0.001, Figures 1C,D).

To verify whether the loss of dopaminergic neurons in the SN caused by 6-OHDA may be associated with glucose homeostasis disturbances; blood sugar analysis was performed to identify the possible alterations associated with dopamine loss. Considering that glucose intolerance may reflect an elevation in blood glucose, glucose tolerance was assessed on the 4th and 6th weeks of the experiment. At 30 min after i.p. injecting a glucose solution, the blood glucose level of the rats treated with 6-OHDA was significantly increased compared with that of the control rats at the 6th week (*n* = 10, *p* < 0.01, Figure 1F), which revealed attenuated glucose tolerance in the 6-OHDA rats. The blood glucose values at 30 min were 0.02 ± 0.00 (mmol/L/kg) in the control group and 0.03 ± 0.00 (mmol/L/kg) in the 6-OHDA group after normalization to body weight. However, this difference between the two groups was not observed at the 4th week (Figure 1E).

### Decreased Expression of D2 and TH in the PVN of 6-OHDA-Induced Rats

**FIGURE 2:**
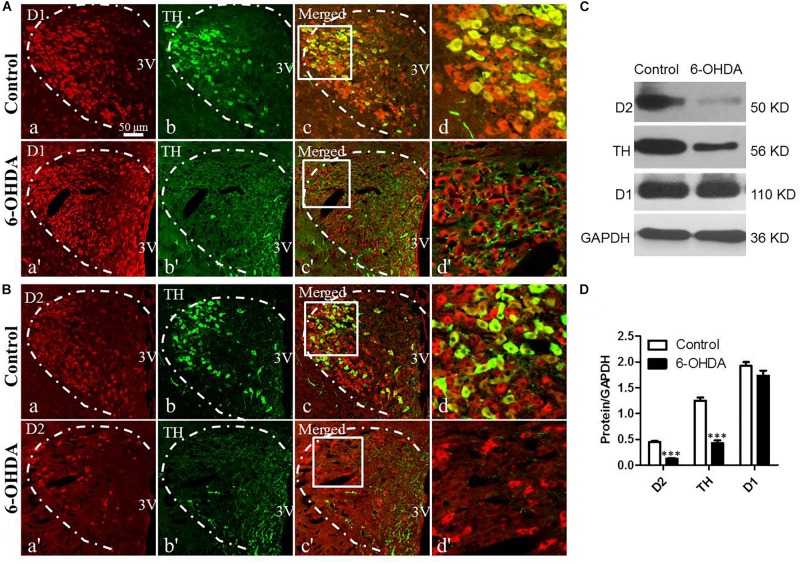
Decreased expression of D2 and TH in the PVN of 6-OHDA-induced rats. **(A,B)** Double-labeling immunofluorescence of D1 (Aa and Aa′) or D2 (Ba and Ba′) (red) and TH (green, Control group: Ab and Bb; 6-OHDA group: Ab′ and Bb′) in the PVN neurons (white dashed frame) of the control and 6-OHDA groups; **(d,d′)** are the enlargement of the white frame in **(c,c′)**, respectively. Scale bar is 50 μm; **(C)** Representative western blot of TH, D1, and D2 in the hypothalamus of control and 6-OHDA rats. **(D)** Summary histogram of **C**, ^∗∗∗^*p* < 0.001 (*n* = 6). 3v: Third ventricle.

To evaluate the effect of the degeneration of dopaminergic neurons in the SN on the DRs in the PVN and their relationship with dopaminergic fibers, double IF and western blot were used. Both TH-IR neurons and nerve fibers were observed in the PVN (Figures 2Ab,Bb). D1-IR and D2-IR neurons were also detected in the PVN (Figures 2Aa,Ba), which was consistent with our previous study (Ran et al., 2019). Double labeling experiments showed that TH-IR neurons were also D1/D2-IR (Figures 2Ac,d,Bc,d), while the D1/D2-IR neurons were surrounded by the majority of the TH-IR fibers (Figures 2Ad,d′,Bd,d′) in the PVN. However, nearly all the TH-IR neurons were lost in the 6-OHDA rats, and only TH-IR fibers were left (Figures 2Ab,b′,c,c′,Bb,b′,c,c′). In addition, after the destruction of dopamine in the SN, the expression of D2 also significantly decreased (Figures 2Ba,a′), whereas there was no change in D1 expression (Figures 2Aa,a′). The western blot results were consistent with the IF results, which further confirmed the reality of the expression of TH and D1/D2 (Figures 2C,D).

### Increased Expression of CRH, ACTH, and CORT in the HPA System of 6-OHDA Rats

**FIGURE 3:**
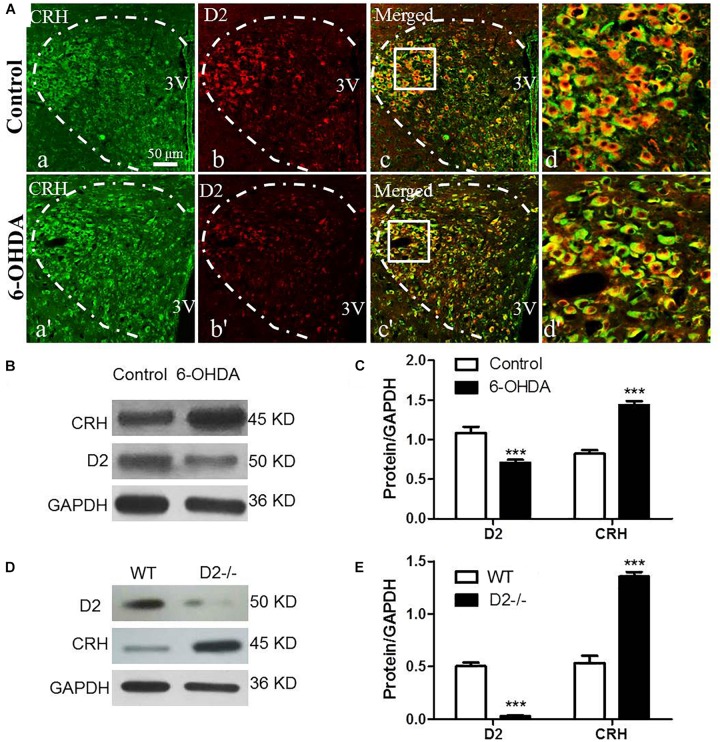
Upregulated CRH and downregulated D2 in the PVN of 6-OHDA-induced rats. **(A)** Double-labeling immunofluorescence of CRH (green: a,a′) and D2 (red: b,b′) in the PVN neurons (white dashed frame) of the control and 6-OHDA groups; **d** and **d′** are the enlargement of the white frame in **c** and **c′**, respectively. Scale bar is 50 μm; **(B)** Representative western blot of D2 and CRH in the hypothalamus of control and 6-OHDA rats. **(C)** Summary histogram of B (*n* = 6). **(D)** Representative western blot of D2 and CRH in the hypothalamus of wild type (WT) and D2−/− mice. **(E)** Summary histogram of D. ^∗∗∗^*p* < 0.001 (*n* = 6).

The CRH-related HPA axis in the rat consists of CRH in the PVN, ACTH in the pituitary gland and glucocorticoids (mainly CORT) in the adrenal gland, which are eventually released into the blood. To investigate the role of the HPA axis in the decreased glucose tolerance of 6-OHDA rats, hypothalamus, pituitary gland, and blood samples were collected to perform histological and immunological tests. As illustrated in Figure 3A, compared with control rats, the 6-OHDA rats had enhanced expression of CRH in the PVN (Figure 3A), and this result was further confirmed by western blot analysis (Figures 3B,C). To further confirm the correlation of D2 expression with CRH in the PVN, we employed D2 knock-out mice. We found that the CRH expression in the PVN was significantly increased in the D2 knock-out mice compared with the control mice (Figures 3D,E). Moreover, morphological analysis also revealed the coexpression of CRH and D2 in the PVN (Figure 3Ad). As expected, the contents of ACTH in the pituitary gland and CORT in the blood were significantly increased by radioimmunoassay (Table 3).

**TABLE 3:** Hormone contents in blood and brain tissues of rats (mean ± SD, *n* = 6).

**Group**	**Pituitary gland**	**Blood**
		
	**ACTH (pg.ml^–1^)**	**CORT (ng.ml^–1^)**
Control	2768.91 ± 290.57	82.07 ± 12.1
6-OHDA	3363.76 ± 316.56^*^	124.61 ± 19.1^*^

ACTH, adrenocorticotropic hormone; CORT, corticosterone; ^*^p < 0.05.

## Discussion

In the present study, we demonstrated that the central administration of 6-OHDA attenuated glucose tolerance and activated the HPA system, as evidenced by the upregulated expression of CRH in the PVN, the increased content of ACTH in the pituitary gland and the increased content of CORT in the blood in the 6-OHDA group compared with the control group. In addition, we showed that D2 was expressed on CRH-IR neurons and that when the SN was destroyed, D2 expression was downregulated. Furthermore, the D2 knock-out mice had significantly upregulated CRH in the PVN.

The dysregulation of glucose metabolism can occur early in the course of sporadic PD (Dunn et al., 2014). Several studies have suggested that type 2 diabetes mellitus (T2DM) is a risk factor for the development of PD (Hu et al., 2007; Pagano et al., 2018). An association between a gene involved in glucose metabolism control and PD was found in genetic microarray studies (Zheng et al., 2010). In addition, a high prevalence of T2DM was also reported in patients with PD (Pressley et al., 2003). The present study showed decreased glucose tolerance after the central destruction of SN dopaminergic neurons by 6-OHDA, which was consistent with the aforementioned studies and provided animal-based experimental evidence that the central dopaminergic system could influence glucose metabolism. However, the underlying pathophysiological mechanisms remain unclear.

Parkinson’s disease is known to damage several brain areas and may affect the regions involved in metabolic control, such as the hypothalamus (Dayan et al., 2018) and especially the PVN (Mann and Yates, 1983; Jellinger, 1991). We previously reported that SN dopaminergic neurons directly project to the PVN by the neural tracing method (Wang et al., 2014). Recently, we further confirmed the expression and distribution of DRs in the PVN (Ran et al., 2019). In the present study, the coexpression of D2 and CRH in the same neurons and the surrounding TH-IR fibers provide the morphological basis for our speculation that the SN in the midbrain could regulate the function of CRH-IR neurons in the PVN of the hypothalamus via DRs. D1 and D2 are the representative subtypes of the DR family that are G protein-coupled receptors. D1 activates adenylyl cyclase and upregulates the intracellular cAMP signaling pathway, whereas D2 inhibits the adenylyl cyclase and downregulates cAMP levels (Baldessarini and Tarazi, 1996). In the present study, after the central destruction of SN dopamine by 6-OHDA, downregulated D2 was observed in the PVN and D1 was not altered, which indicates that the inhibition of CRH-IR neurons by the D2 signaling pathway was attenuated. In other words, the synthesis of CRH in the PVN was upregulated when D2 was downregulated, which was further confirmed in D2 knock-out mice. The altered expression of D2 and CRH in the PVN provides clues for exploring HPA axis-based glucose metabolism in PD.

The HPA neuroendocrine system consists of three populations of cells, and each population of cells secretes specialized hormones. The neurons in the PVN secrete CRH (Hauger et al., 2003), the endocrine cells (corticotrophs) in the anterior pituitary secrete ACTH (Allen and Sharma, 2019), and the endocrine cells primarily in the zona fasciculata of the adrenal cortex secrete the glucocorticoid hormones cortisol and/or CORT. CORT is secreted into the systemic circulation and affects the cells throughout the body (Spencer and Deak, 2017). Glucocorticoid hormones can increase the synthesis of hepatic glycogen, reduce the utilization and decomposition of glycogen in tissues, and elevate blood sugar (Si et al., 2015). In the present study, we observed increased CRH expression in the PVN, elevated ACTH in the pituitary and increased CORT content in the blood in 6-OHDA rats. The increased CORT level in the 6-OHDA rat is possibly due to the altered HPA axis activity because of the enhanced CRH expression in the PVN and increased ACTH content in pituitary (Spencer and Deak, 2017). This hypothesis means that the dopamine in the SN could regulate the function of the HPA axis via DRs in the PVN through the SN-PVN dopaminergic nerve pathway.

The interaction between neurodegeneration and metabolic disorders is complex and is influenced by many factors, which in turn make it difficult to define causal factor(s) (Yang et al., 2017; Biosa et al., 2018; De Pablo-Fernandez et al., 2018). In the present study, it seemed that the decreased glucose tolerance was due to the loss of dopaminergic neurons in the SN, which suggests that neurodegeneration occurred first and then influenced metabolism. Patients with PD have glucose intolerance (Lipman et al., 1974). Elevated plasma cortisol is strongly associated with glucose intolerant (Reynolds et al., 2001). It is well established that cortisol excess causes insulin resistance in man (Conn and Fajans, 1956; Rooney et al., 1993), which in turn impairs the ability of insulin to suppress glucose production and glucose utilization resulted from glucocorticoid excess (Munck, 1971). It has been reported that cortisol-induced insulin resistance is due to a decrease in both hepatic and extrahepatic sensitivity to insulin which can be explained on the basis of post-receptor defect (Rizza et al., 1982). Therefore, in our present study, the observed glucose intolerance accompanied by the activated HPA axis in 6-OHDA rats could be associated with the insulin resistance. However, CORT induces the direct negative feedback of corticotrophs in the anterior pituitary and in the CRH neurons in the PVN. The activity of the HPA axis is directly and indirectly controlled by various neural activities present throughout the forebrain and brainstem. Therefore, the underlying mechanism of the abnormality of the HPA axis in patients with PD or in PD animal models remains to be elucidated.

## Data Availability

All datasets generated for this study are included in the manuscript and/or the supplementary files.

## Ethics Statement

Every procedure was approved by the Animal Care and Use Committee of Capital Medical University and was conducted according to the established guidelines of the National Institutes of Health (NIH, United States).

## Author Contributions

J-XZ designed the research. LZ and X-RR performed the experiments. FH and G-WL helped the data analysis and provided the technical support. LZ wrote the manuscript. J-XZ revised the manuscript.

## Conflict of Interest Statement

The authors declare that the research was conducted in the absence of any commercial or financial relationships that could be construed as a potential conflict of interest.

## References

[B1] Allen M. J. Sharma S. (2019). *Physiology, Adrenocorticotropic Hormone (ACTH).* Treasure Island: StatPearls Publishing LLC. 29763207

[B2] Baldessarini R. J. Tarazi F. I. (1996). “Brain dopamine receptors: a primer on their current status, basic and clinical.” *Harv. Rev. Psychiatry* 3 301–325. 9384962 10.3109/10673229609017200

[B3] Biosa A. Outeiro T. F. Bubacco L. Bisaglia M. (2018). “Diabetes Mellitus as a risk factor for parkinson’s disease: a molecular point of view.” *Mol. Neurobiol.* 55 8754–8763. 10.1007/s12035-018-1025-9 29594935

[B4] Conn J. W. Fajans S. S. (1956). Influence of adrenal cortical steroids on carbohydrate metabolism in man. *Metabolism* 5 114–127. 13296872

[B5] Dayan E. Sklerov M. Browner N. (2018). Disrupted hypothalamic functional connectivity in patients with PD and autonomic dysfunction. *Neurology* 90 e2051–e2058. 10.1212/WNL.0000000000005641 29728527

[B6] De Pablo-Fernandez E. Courtney R. Holton J. L. Warner T. T. (2017). “Hypothalamic alpha-synuclein and its relation to weight loss and autonomic symptoms in Parkinson’s disease.” *Mov. Disord.* 32 296–298. 27892607 10.1002/mds.26868

[B7] De Pablo-Fernandez E. Goldacre R. Pakpoor J. Noyce A. J. Warner T. T. (2018). Association between diabetes and subsequent Parkinson disease: a record-linkage cohort study. *Neurology* 91 e139–e142. 10.1212/WNL.0000000000005771 29898968

[B8] Dunn L. Allen G. F. Mamais A. Ling H. Li A. Duberley K. E. (2014). “Dysregulation of glucose metabolism is an early event in sporadic Parkinson’s disease.” *Neurobiol. Aging* 35 1111–1115. 10.1016/j.neurobiolaging.2013.11.001 24300239 PMC3969149

[B9] Hauger R. L. Grigoriadis D. E. Dallman M. F. Plotsky P. M. Vale W. W. Dautzenberg F. M. (2003). International Union of Pharmacology. XXXVI. current status of the nomenclature for receptors for corticotropin-releasing factor and their ligands. *Pharmacol. Rev.* 55 21–26. 12615952 10.1124/pr.55.1.3

[B10] Hu G. Jousilahti P. Bidel S. Antikainen R. Tuomilehto J. (2007). Type 2 diabetes and the risk of Parkinson’s disease. *Diabetes Care* 30 842–847. 17251276 10.2337/dc06-2011

[B11] Jackson-Lewis V. Blesa J. Przedborski S. (2012). “Animal models of Parkinson’s disease.” *Parkinsonism Relat. Disord.* 18 (Suppl. 1), S183–S185. 10.1016/S1353-8020(11)70057-8 22166429

[B12] Jellinger K. A. (1991). Pathology of Parkinson’s disease. changes other than the nigrostriatal pathway. *Mol. Chem. Neuropathol.* 14 153–197. 1958262 10.1007/BF03159935

[B13] Lipman I. J. Boykin M. E. Flora R. E. (1974). Glucose intolerance in Parkinson’s disease. *J. Chronic. Dis.* 27 573–579. 4436423 10.1016/0021-9681(74)90031-9

[B14] Lu J. Montgomery B. K. Chatain G. P. Bugarini A. Zhang Q. Wang X. (2018). “Corticotropin releasing hormone can selectively stimulate glucose uptake in corticotropinoma via glucose transporter 1.” *Mol. Cell. Endocrinol.* 470 105–114. 10.1016/j.mce.2017.10.003 28986303 PMC5882598

[B15] Mann D. M. Yates P. O. (1983). “Pathological basis for neurotransmitter changes in Parkinson’s disease.” *Neuropathol. Appl. Neurobiol.* 9 3–19. 6133229 10.1111/j.1365-2990.1983.tb00320.x

[B16] Munck A. (1971). “Glucocorticoid inhibition of glucose uptake by peripheral tissues: old and new evidence, molecular mechanisms, and physiological significance.” *Perspect. Biol. Med.* 14 265–269. 5546253 10.1353/pbm.1971.0002

[B17] Oh T. J. Shin J. Y. Kang G. H. Park K. S. Cho Y. M. (2013). “Effect of the combination of metformin and fenofibrate on glucose homeostasis in diabetic Goto-Kakizaki rats.” *Exp. Mol. Med.* 45:e30. 10.1038/emm.2013.58 23827952 PMC3731660

[B18] Pagano G. Polychronis S. Wilson H. Giordano B. Ferrara N. Niccolini F. (2018). Diabetes mellitus and Parkinson disease. *Neurology* 90 e1654–e1662. 10.1212/WNL.0000000000005475 29626177

[B19] Pfeiffer R. F. (2016). “Non-motor symptoms in Parkinson’s disease.” *Parkinsonism. Relat. Disord.* 22 (Suppl. 1), S119–S122. 10.1016/j.parkreldis.2015.09.004 26372623

[B20] Pressley J. C. Louis E. D. Tang M. X. Cote L. Cohen P. D. Glied S. (2003). The impact of comorbid disease and injuries on resource use and expenditures in parkinsonism. *Neurology* 60 87–93. 12525724 10.1212/wnl.60.1.87

[B21] Ran X. Yang Y. Meng Y. Li Y. Zhou L. Wang Z. (2019). Distribution of D1 and D2 receptor- immunoreactive neurons in the paraventricular nucleus of the hypothalamus in the rat. *J. Chem. Neuroanat.* 98 97–103. 10.1016/j.jchemneu.2019.04.002 31018158

[B22] Reynolds R. M. Walker B. R. Syddall H. E. Whorwood C. B. Wood P. J. Phillips D. I. (2001). “Elevated plasma cortisol in glucose-intolerant men: differences in responses to glucose and habituation to venepuncture.” *J. Clin. Endocrinol. Metab.* 86 1149–1153. 11238500 10.1210/jcem.86.3.7300

[B23] Rizza R. A. Mandarino L. J. Gerich J. E. (1982). “Cortisol-induced insulin resistance in man: impaired suppression of glucose production and stimulation of glucose utilization due to a postreceptor detect of insulin action.” *J. Clin. Endocrinol. Metab.* 54 131–138. 7033265 10.1210/jcem-54-1-131

[B24] Rooney D. P. Neely R. D. Cullen C. Ennis C. N. Sheridan B. Atkinson A. B. (1993). “The effect of cortisol on glucose/glucose-6-phosphate cycle activity and insulin action.” *J. Clin. Endocrinol. Metab.* 77 1180–1183. 8077310 10.1210/jcem.77.5.8077310

[B25] Si M. W. Yang M. K. Fu X. D. (2015). “Effect of hypothalamic-pituitary-adrenal axis alterations on glucose and lipid metabolism in diabetic rats.” *Genet. Mol. Res.* 14 9562–9570. 10.4238/2015.August.14.19 26345889

[B26] Spencer R. L. Deak T. (2017). “A users guide to HPA axis research.” *Physiol. Behav.* 178 43–65. 10.1016/j.physbeh.2016.11.014 27871862 PMC5451309

[B27] Stern M. B. Lang A. Poewe W. (2012). “Toward a redefinition of Parkinson’s disease.” *Mov. Disord.* 27 54–60. 22252891 10.1002/mds.24051

[B28] Wang Z. Y. Lian H. Cai Q. Q. Song H. Y. Zhang X. L. Zhou L. (2014). No direct projection is observed from the substantia nigra to the dorsal vagus complex in the rat. *J. Parkinsons. Dis.* 4 375–383. 10.3233/JPD-130279 24613863

[B29] Yang Y. W. Hsieh T. F. Li C. I. Liu C. S. Lin W. Y. Chiang J. H. (2017). Increased risk of Parkinson disease with diabetes mellitus in a population-based study. *Medicine* 96:e5921. 10.1097/MD.0000000000005921 28099356 PMC5279101

[B30] Zheng B. Liao Z. Locascio J. J. Lesniak K. A. Roderick S. S. Watt M. L. (2010). PGC-1alpha, a potential therapeutic target for early intervention in Parkinson’s disease. *Sci Transl Med* 2:52ra73. 10.1126/scitranslmed.3001059 20926834 PMC3129986

[B31] Zheng L. Wang Z. Li X. Song J. Hong F. Lian H. (2011). Reduced expression of choline acetyltransferase in vagal motoneurons and gastric motor dysfunction in a 6-OHDA rat model of Parkinson’s disease. *Brain Res.* 1420 59–67. 10.1016/j.brainres.2011.09.006 21955729

[B32] Zheng Z. Travagli R. A. (2007). Dopamine effects on identified rat vagal motoneurons. *Am. J. Physiol. Gastrointest. Liver Physiol.* 292 G1002–G1008. 17170022 10.1152/ajpgi.00527.2006 PMC3070949

[B33] Zhou L. Wang Z. Y. Lian H. Song H. Y. Zhang Y. M. Zhang X. L. (2014). Altered expression of dopamine receptors in cholinergic motoneurons of the hypoglossal nucleus in a 6-OHDA-induced Parkinson’s disease rat model. *Biochem. Biophys. Res. Commun.* 452 560–566. 10.1016/j.bbrc.2014.08.104 25172664

